# Prevalence of Wheat Associated *Bacillus* spp. and Their Bio-Control Efficacy Against *Fusarium* Root Rot

**DOI:** 10.3389/fmicb.2021.798619

**Published:** 2022-03-03

**Authors:** Shah Mulk, Abdul Wahab, Humaira Yasmin, Saqib Mumtaz, Hamed A. El-Serehy, Naeem Khan, Muhammad Nadeem Hassan

**Affiliations:** ^1^Department of Biosciences, COMSATS University Islamabad, Islamabad, Pakistan; ^2^Department of Zoology, College of Science, King Saud University, Riyadh, Saudi Arabia; ^3^Department of Agronomy, Institute of Food and Agricultural Sciences, Florida University, Gainesville, FL, United States

**Keywords:** *Bacillus* spp., cropping system, prevalence, root rot, wheat-rice

## Abstract

*Bacillus* spp. are the most prevalent group of bacteria in nature. Their prevalence depends upon multiple factors, namely, sporulation, antagonism, and production of secondary metabolites. The development of an eco-friendly approach to cope with edible crops diseases is very substantial for humans. In the present study, 658 isolates were obtained from wheat grown in the wheat rice cropping system and tested for their antagonistic activity against four wheat root rot pathogens, namely, *Fusarium oxysporum, Fusarium moniliforme, Macrophomina phaseolina*, and *Rhizoctonia solani*. Out of 658, 106 isolates were found antagonistic to either single or multiple fungi. Out of 106 antagonistic bacteria, 62 (23%) were rhizospheric, 28 (14%) were root endospheric, and 16 (9%) were leaf endospheric. Based on mean inhibition against all fungi, the bacterial strains SM-39 and SM-93 showed maximum antagonistic activity. The 16S rRNA gene analysis revealed that most of the antagonistic bacteria exhibiting ≥48% antagonism were *Bacillus* spp. (98%), except two were *Klebsiella* spp. (2%). The bacterial strains exhibited phylogenetic lineage with the type strains of the respective genus based on the 16S rRNA gene sequences. In the net house experiment, *Bacillus velezensis* (SM-39) and *Bacillus cabrialesii* (SM-93) significantly suppressed *Fusarium* root rot severity in wheat (42–62%). Plants treated with these strains had lower electrolytic leakage (29–36%), as compared to untreated (44%). Relative water content was much higher (46–58%) for plants inoculated with these strains. These antagonistic strains also considerably colonized the wheat rhizosphere with a cell population of 5.8–6.9.log CFU/g of soil. The rhizosphere of wheat grown in the wheat-rice cropping system could be the potential habitat of effective biocontrol agents.

## Introduction

Phytopathogenic fungi affect the field crops adversely and pose a serious threat to agriculture. They not only decrease the crop yield but also deteriorate their quality. They cause destructive damage to crops, leading to economic losses of 1 billion dollars globally (Volova et al., 2018). Among various pathogens, root rot fungi such as *Macrophomina phaseolina, Fusarium oxysporum, Fusarium moniliforme*, and *Rhizoctonia solani* are the most devastating pathogens of cereals including wheat (Islam et al., 2013). Their infection coupled with the prolonged drought conditions poses a serious threat to global food security (Fira et al., 2018).

Biological control of phytopathogens using plant growth-promoting rhizobacteria (PGPR) is an eco-friendly strategy (Parry et al., 2016; Dangi et al., 2017; Khanna et al., 2019a). A plethora of literature is available on the activity of PGPR (Khanna et al., 2019b; Sharma et al., 2020) but their commercial application is still limited. The factors responsible for their commercial limitation include lack of consistent field efficacy and poor shelf life (Valente et al., 2020).

The field performance of PGPR is typically inconsistent because of sub-optimal rhizosphere colonization and promiscuous host-specificity (Martiny et al., 2006). The higher persistence of certain microbes in the rhizosphere is a key factor that determines their consistent efficacy. Sometimes, the efficient rhizobacteria lose their efficacy due to competition with the soil microbiota. The antagonistic potential of microbes plays an important role in determining the spatial structuring of bacterial communities. This has also been formerly verified in different bacterial communities such as endophytic bacteria, whose antagonism has been described as one of the reasons for structuring the community within different plant compartments (Chiellini et al., 2019).

Rhizobacteria-host adaptation is a complex phenomenon and is regulated by multiple factors including bacterial type, host genotype, and rhizosphere environment (Brader et al., 2014). As the rhizobacteria are recognized by a certain host, crosstalk of the signaling molecules is initiated (Verma et al., 2021). The signaling also includes the chemotactic movements of microbes regulated by the root exudates of plants (Abid et al., 2018). The occurrence of certain bacterial communities is highly influenced by biotic and abiotic factors. In agriculture niches/habitats, various cultural practices like irrigation, fertilization, pathogen infestation, and cropping pattern highly influence the diversity and activity of the microbes inhabiting certain rhizosphere.

Wheat (*Triticum aestivum*) being a staple food is grown worldwide under different cropping systems such as fallow-wheat, wheat-maize, and wheat-rice (Koondhar et al., 2018). Wheat-rice is the most promising cropping pattern as it involves the rotation of economically important crops. The bacterial communities of multiple cropping systems have been reported as diverse than that of mono-cropping systems (Bhattacharyya and Jha, 2012).

Plant growth-promoting rhizobacteria belong to diverse genera such as *Arthrobacter, Azospirillum, Bacillus, Burkholderia, Chromobacterium, Caulobacter, Erwinia, Enterobacter, Flavobacterium, Pseudomonas*, and *Klebsiella*. The distribution of rhizobacterial communities varies with the host species, genotype, growth stage, location, and growing practices (Hakim et al., 2018). Although various studies have been conducted to know the diversity of rhizospheric bacteria using culture-dependent and independent techniques (Qaisrani et al., 2019), less attention has been paid to the prevalence of antagonistic bacteria in the rhizosphere of crops grown at the commercial scale in the specific cropping system. Based on these facts, this study aimed to recruit the antagonistic bacteria effective against wheat root rot and identify the most prevalent antagonistic genera associated with the wheat grown in the wheat-rice cropping system.

## Materials and Methods

### Survey and Sampling

A survey was conducted to identify the major areas practicing the wheat-rice cropping system in Punjab, Pakistan. Representative districts, namely, Gujranwala, Gujrat, Sialkot, Lahore, Kasur, Sheikhupura, and Nankana Sahib, within these areas were selected for sampling. Among each district, two types of zones were identified based on their mode of irrigation, i.e., canal and tube well. Two to three fields were randomly identified in a radius of 2 km of the respective zone. Three to four plants (healthy and diseased) with variable phenotypes were sampled randomly from different corners of the field. The plants were generally uprooted manually. However, a mini spade (6′′ × 9′′) with a 1-m handle was used to uproot the plants from moisture deficient soil to minimize the risk of root damage. The spade was surface sterilized with 70% ethanol before using it on the other samples. The uprooted plants were mixed to make the representative sample of each category. The representative samples were tagged, placed in paper bags, brought to the laboratory at 4°C, and processed immediately.

### Rhizosphere Soil and Physiochemical Analysis

Rhizospheric soil was carefully removed from the root surfaces by vigorously shaking on sterilized aluminum foil. The soil was analyzed for different physiochemical parameters such as organic matter, electrical conductivity, pH, total nitrogen, phosphorous, potassium, sodium, and sodium absorption ratio (SAR) at Fatima Sugar Mills Limited, Kot Addu, Punjab, Pakistan.

### Isolation of Bacteria

The bacterial isolates were obtained from the root rhizosphere, root, and leaf endosphere of plants by the serial dilution method (Ullah et al., 2020; Riaz et al., 2021). For endophytic isolation, the roots/leaves were washed with tap water to remove surface impurities and surface sterilized with 0.1% mercuric chloride (HgCl_2_) (Rais et al., 2016). Briefly, the plant tissues were dipped in solution (0.1% HgCl_2_) for 1–2 min, washed twice with sterile distilled water, dried, and crushed in a sterilized mortar pestle.

The rhizosphere soil or tissue homogenate (1 g) was suspended in 9 mL of sterile saline (0.9% w/v) and diluted serially (10^–1^–10^–8^). The bacterial colonies were obtained by spreading each dilution (100 μL) on Luria-Bertani (LB) agar and incubating at 37 ± 2°C overnight. The bacterial colonies appearing on agar were differentiated based on their morphology like shape, size, margins, and elevations. Different isolates were sub-cultured and preserved in 20% glycerol at −0 ± 2°C for future studies (Rais et al., 2016).

### Fungal Antagonism

Antagonistic activity of bacteria was assessed against wheat root rot fungi such as *F. oxysporum, F. moniliforme, M. phaseolina*, and *R. solani.* Pure fungal cultures were obtained from Applied Microbiology and Biotechnology Laboratory, COMSATS University, Islamabad, Pakistan. A dual culture assay was performed as described in earlier studies (Ullah et al., 2020). Briefly, 5 mm mycelium plug of each fungus (5–7 days old) was kept at the center of the plate containing potato dextrose agar (PDA) and freshly grown bacteria were inoculated at equal distances from each fungus. Sterilized LB broth was used as a control. The plates were incubated at 28 ± 2°C and observed for zone inhibition until the 7th day. The percentage inhibition was calculated by the following formula.


PercentInhibition=[(C-T)/C×100)]

where C, mycelium diameter of fungus taken as control; T, mycelium diameter of fungus treated with bacteria.

### Molecular Identification

The antagonistic bacteria were identified by 16S rRNA gene analysis. Genomic DNA was extracted by CTAB method (Moore et al., 1999). The extracted DNA was qualitatively analyzed on agarose gel (1% W/V) pre-stained with ethidium bromide and electrophoresed in 1× TBE buffer at 100 V for 30–40 min.

DNA bands were visualized in the Gel documentation system (Biometra, Germany) and their quality was compared with that of the DNA ladder (Thermo Scientific). The 16S rRNA gene was amplified by using the primers P1 and P6 (Tan et al., 1997). A 50 μL PCR reaction consisted of PCR water (31 μL), 5 μL Taq buffer (10x), 4 μL MgCl_2_ (25 mM), 3 μL dNTPs (10 mM), 2 μL of each primer (100 pM), 0.5 μL Taq polymerase (500 U), and 2.5 μL template DNA (25 ng/μL). The reaction was amplified in a thermal cycler (Applied Biosystems, United States) using the cycling conditions, namely, initial denaturation (95°C for 5 min), 25 cycles x (denaturation at 95°C for 1 min, annealing at 56°C for 1 min, extension at 72°C for 1.5 min) and final extension at 72°C for 5 min. The PCR products were visualized by gel electrophoresis (as described above) and purified by using PCR purification kit (Thermo Scientific).

### Sequencing, Nucleotide Accession Number, and Phylogenetic Analysis

The purified PCR products of 16S rRNA gene were sequenced by Macrogen Incorporation, South Korea, and analyzed for homology with that of the closest type strain available at EZ taxon^1^ (Kumar et al., 2016). The 16S rRNA gene sequence of each strain was submitted to the gene bank^2^ under its respective accession number. For phylogenetic analysis, 16S rRNA genes of respective type strains were accessed from EZ taxon.^3^ These sequences were oriented in the same direction, i.e., 5′-3′, saved in FASTA format and uploaded on MEGA X software (Molecular Evolutionary Genetics Analysis X). The sequences were aligned and trimmed to the proper length to get conserved regions. These trimmed sequences were then converted into MEGA X format and saved for phylogenetic tree construction. The phylogeny was created by using the neighbor joining phylogenetic tree option in MEGA X software (Afzal et al., 2019). Evolutionary distances were calculated by using the maximum composite likelihood model and were in the units of the number of base substitutions per site.

### *In planta* Biocontrol Activity

Bio control efficacy of the *Bacillus* spp., i.e., *Bacillus velezensis* and *Bacillus cabrialesii* showing the best *in vitro* inhibition (60–63%) against root rot pathogens was assessed on wheat against *Fusarium* root rot. The experiment was carried out at COMSATS University, Islamabad, Pakistan (33.65°N, 73.16°) in the net house conditions during the natural growing season of wheat, i.e., November–December (2019–2020) (humidity = 65% and average temperature = 15.8°C). The seeds of wheat varieties, i.e., Galaxy and Sahar were obtained from National Agriculture Research Centre (NARC), Islamabad, Pakistan. There were five treatments, with three replications per treatment, namely, untreated plants (1), fungicide (Dimethomorph) (2), SM-39 (*B. velezensis*) (3), SM-93 (*B. cabrialesii*) (4), and a consortium of SM-39, SM-93 (5). The experiment was repeated twice during the years 2019–2020.

The soil [clay loam, phosphorous (12–14 mg/kg), total nitrogen (0.03–0.05%); organic matter (0.5–0.7%), and pH (7.6–7.8)] was obtained from a wheat field, autoclaved twice, filled in pots (17 cm × 21 cm), and fertilized with nitrogen (120 Kg/ha), phosphorous (100 Kg/ha), and potassium (60 Kg/ha) (Ramzani et al., 2016), (Ullah et al., 2020). The upper layer of soil (2–3 inches) was infested with the root rot pathogen *F. moniliforme* (Jaber, 2018). The fungus was grown on PDA for 7 days at 30 ± 2°C, scrapped with the spatula, and suspended in sterile water. The spore suspension was mixed with the upper layer of soil at the rate of 10^5^ spores per g of soil.

The seeds were surface sterilized with 0.1% sodium hypo chloride (NaClO), dressed with bacterial cell suspension (4 × 10^9^ CFU/mL), carboxy methyl cellulose (1% w/v), fungicide (Dimethomorph 90 g w/w) and sown in pots. Two plants were maintained in each pot and five plants were included in each replication. All the agronomic practices were followed as per standard recommendations (Ullah et al., 2020).

#### Disease Assessment

The disease was examined on the 60th day of seed sowing. Fifteen plants/treatment were randomly selected, uprooted manually, and assessed for root rot disease on a scale (0–5), i.e., 0 = healthy (0%), 1 = very slight browning (10–20%), 2 = slight to moderate browning (20–40%), 3 = moderate (40–60%), 4 = severe (>60%), and 5 = completely necrotic or dead (100%). Disease severity was determined by using the formula as described by Wildermuth and McNamara (1994).


$$\text{Disease}\,\text{severity}=\frac{\Sigma \,\text{xf}}{\text{n}\,\Sigma \,\text{f}}\times 100$$

where x is the value of disease score, n is the value of the highest disease score, and f is the number of plants for each score.

#### Relative Water Content and Electrolytic Leakage

The relative water content of wheat leaves was determined as described by Guerfel et al. (2009). The fresh leaves (4–5) per replication were randomly selected, chopped, and pooled. The fresh leaves (0.5 g) were soaked in 100 mL distilled water at room temperature (27°C) for 24 h to record their turgid weight. Then, these leaves were kept at 70°C in a dry oven until the appearance of constant weight. Relative water content was calculated according to the formula used by Riaz et al. (2021).


RWC(%)=FW-DW/TW-DW×100

where FW, fresh weight; DW, dry weight; and TW, turgid weight.

The electrolytic leakage of leaves was determined by the method of Ekmekci and Terzioglu (2005). Briefly, the fresh leaves were sliced and placed in deionized water (20 mL) under dark conditions for 24 h to measure the initial electrical conductivity (EC_1_) at room temperature with a conductivity meter. The leaves were autoclaved to release all the electrolytes. Final electrical conductivity (EC_2_) was measured at room temperature. The electrolytic leakage was calculated by using the following formula:


EL(%)=EC1/EC2×100.

#### Bacterial Colonization on Wheat Roots

The bacterial colonization potential on wheat roots was assessed as described earlier (Ullah et al., 2020). Rhizospheric soil samples from 15 plants per replication (uprooted for diseases assessment as described in section “*In planta* Biocontrol Activity”) were collected and pooled together to make composite samples. The inoculated antagonistic bacteria were isolated by serial dilution method and identified based on their colony morphology and antagonism against respective fungi.

### Statistical Analysis

The numeric data of repeated experiments were pooled and analyzed on the statistical package Statistix 8.1. by applying the analysis of variance (ANOVA). The mean among different treatments were separated by Fisher’s least significant difference (LSD) test at *p* ≤ 0.05. Pearson correlation was determined by using the same statistical package. Venn diagram was constructed by using origin 6.0. Principal component analysis (PCA) was constructed by using software PRIMER 6 and PERMANOVA.

## Results

### Soil Characteristics and Bacterial Isolates

The soil texture was clay loam with organic matter (0.71–0.97%), electrical conductivity (1.0–2.2 ds/m), pH (7.2–8.2), total nitrogen (0.03–0.07%), phosphorous (10.9–13.8 ppm), potassium (170–228 ppm), sodium (146–230 ppm), and SAR (2–3.1) (Table 1). A total of 658 isolates were obtained from wheat grown in rice-wheat cropping system of Punjab, Pakistan. Out of these 658 isolates, 271 were obtained from root rhizosphere, 202 from root endosphere, and 185 from leaf endosphere, respectively.

**TABLE 1:** Physiochemical parameters of soil collected from wheat rhizosphere at various locations.

Location	Irrigation source	pH	EC(ds/m)	Nitrogen (%)	Phosphorus (ppm)	Potassium (ppm)	Organic matter (%)	Sodium (ppm)	SAR
Gujranwala	Tube well	8.2	1.8	0.04	10.9	170	0.71	183	2.8
Gujranwala	Canal	7.8	1.0	0.06	12.2	205	0.84	146	2.1
Gujrat	Tube well	7.9	1.5	0.04	11.5	212	0.77	220	3.1
Gujrat	Canal	7.6	1.2	0.06	12.9	187	0.89	201	2.3
Sialkot	Tube well	8.2	1.4	0.03	11.5	225	0.85	178	2.7
Sialkot	Canal	7.5	1.2	0.05	13.6	210	0.95	170	2.5
Lahore	Tube well	7.8	2.1	0.04	10.6	185	0.82	178	2.6
Lahore	Canal	7.2	1.3	0.07	12.6	210	0.91	152	2.0
Kasur	Tube well	8	1.5	0.04	12.2	208	0.83	230	2.8
Kasur	Canal	7.5	1.2	0.05	13.2	228	0.95	214	2.3
Sheikhupura	Tube well	8	1.3	0.03	11.6	190	0.88	190	2.8
Sheikhupura	Canal	7.2	1.0	0.05	13.5	225	0.97	175	2.5
Nankana Sahib	Tube well	8	2.2	0.04	12.2	208	0.86	225	2.9
Nankana Sahib	Canal	7.6	1.2	0.06	13.8	220	0.91	195	2.4

*EC, electrical conductivity; ppm, parts per million; and SAR, sodium adsorption ratio.*

### Antagonism Against Wheat Root Rot Pathogens

**FIGURE 1:**
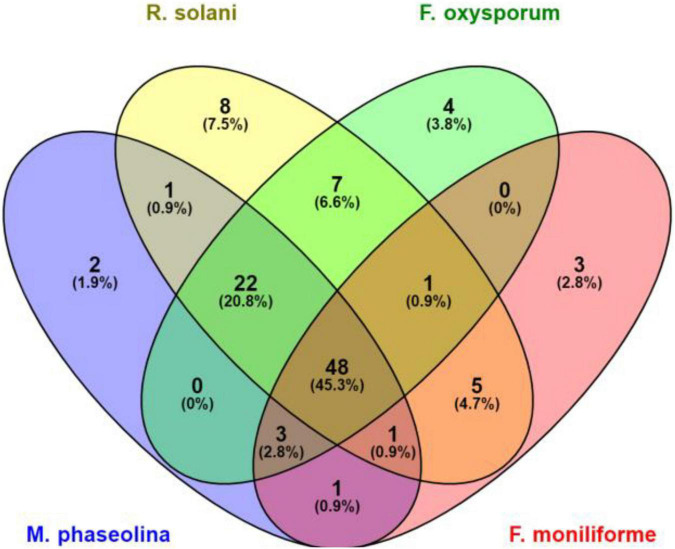
Venn diagram representing the number of antagonists against different root rot fungi. F, *Fusarium*; R, *Rhizoctonia*; and M, *Macrophomina.*

**FIGURE 2:**
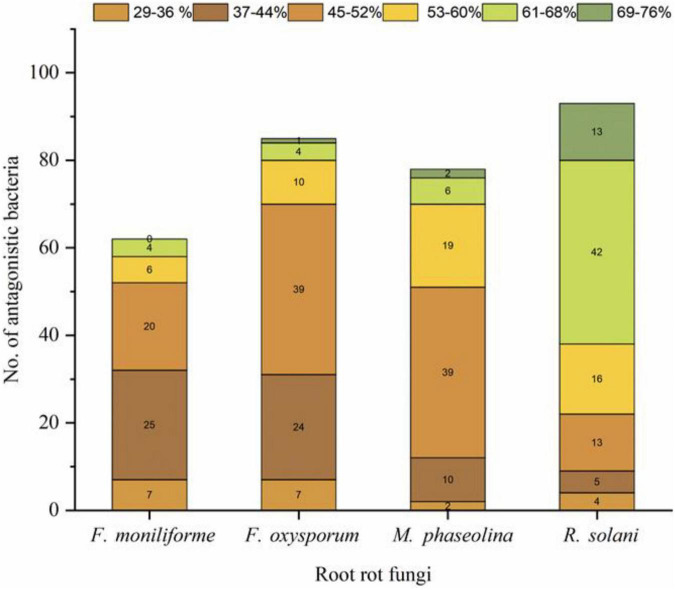
Antagonistic bacteria with variable antagonistic potential (29–76%) against root rot fungi. F, *Fusarium*; R, *Rhizoctonia*; and M, *Macrophomina.*

A total of 658 bacterial isolates associated with wheat root rhizosphere, root endosphere, and leaf endosphere were tested for their antagonistic activity against wheat root rot fungi, namely, *F. moniliforme, F. oxysporum, M. phaseolina*, and *R. solani*. Out of 658 bacterial isolates, 106 isolates were found antagonistic to either single or multiple fungi, namely, *F. oxysporum, F. moniliforme, M. phaseolina*, and *R. solani* (Supplementary Table 1). Out of 106 antagonistic bacteria, 48 strains antagonized four fungi, namely, *F. oxysporum, F. moniliforme, M. phaseolina*, and *R. solani*. Based on a group of three test fungi, 22 strains antagonized *M. phaseolina, F. oxysporum*, and *R. solani.* Three strains antagonized *F. oxysporum, F. moniliforme*, and *M. phaseolina*, one strain antagonized *F. oxysporum, F. moniliforme*, and *R. solani*, and one strain antagonized *F. moniliforme, M. phaseolina*, and *R. solani*, respectively. Seven strains antagonized two fungi, namely, *R. solani* and *F. oxysporum*, five strains antagonized two fungi, namely, *F. moniliforme* and *R. solani*. One strain antagonized *M. phaseolina* and *F. moniliforme* and one strain antagonized *M. phaseolina* and *R. solani.* Eight strains antagonized only *R. solani*, four strains antagonized *F. oxysporum*, three strains antagonized *F. moniliforme* and two strains antagonized only *M. phaseolina* (Figure 1). *R. solani* was antagonized by a maximum number of strains (93), followed by *F. oxysporum* (85), *M. phaseolina* (78), and *F. moniliforme* (62), respectively. The potential of antagonistic bacteria against each fungus was highly variable (29–76%). In the case of *R. solani*, the maximum number of strains (42) showed antagonism between 61 and 68%. The maximum number of strains (39 each) antagonized *F. oxysporum* and *M. phaseolina* between 45 and 52%. While in the case of *F. moniliforme*, the maximum number of strains (25) showed antagonism between 37 and 44% (Figure 2).

### Distribution of Antagonistic Bacteria

**FIGURE 3:**
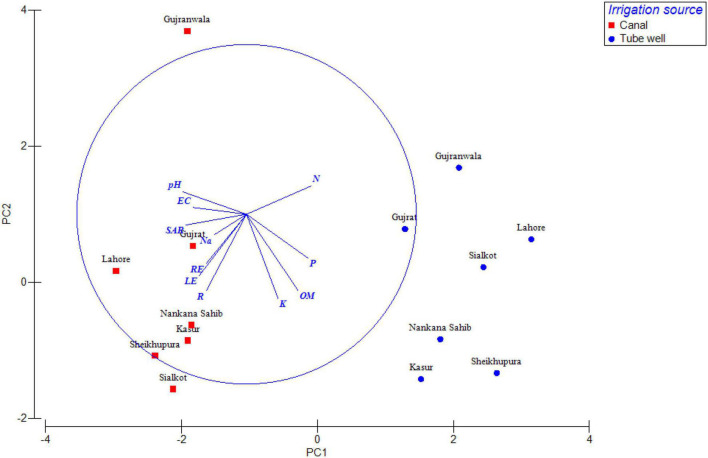
Principal component analysis (PCA) of antagonistic bacteria isolated from tube well/canal irrigated wheat crop. Variables of soil parameters, plant parts, i.e., rhizosphere, root, and leaf endospheres are represented by base vectors. EC, electrical conductivity; SAR, sodium adsorption ratio; P, phosphorous; K, potassium; Na, sodium; N, nitrogen; OM, organic matter; R, rhizosphere; LE, leaf endosphere; and RE, root endosphere.

**FIGURE 4:**
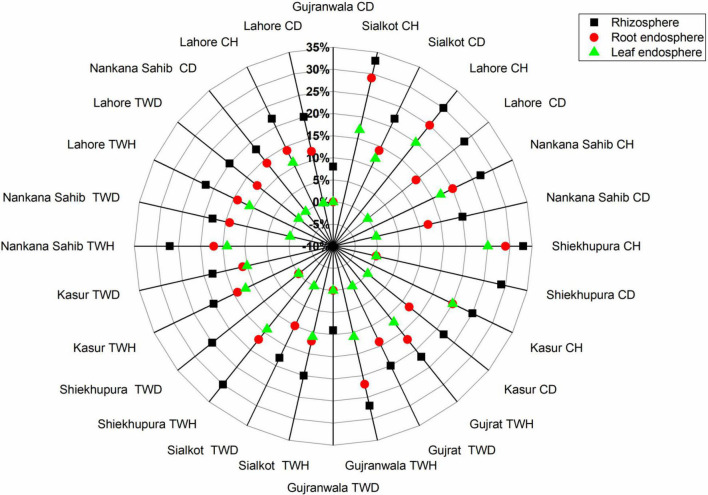
Distribution of percent antagonistic bacteria isolated from rhizosphere, root, and leaf endosphere of wheat irrigated with canal/tube well and grown in wheat-rice cropping system at different locations. TWH, tube well healthy; TWD, tube well disease; CH, canal healthy; and CD, canal disease.

The distribution of antagonistic bacteria was variable in different locations, plant health, irrigation source, and plant parts, i.e., rhizosphere, root, and leaf endospheres. Based on location, highest antagonistic bacteria (29%) were found in Sheikhupura followed by Sialkot (26%), Lahore (25%), Kasur (22%), Gujrat (21%), and Gujranwala (20%), respectively. In the case of irrigation source, the highest antagonism was found in canal irrigated (3–29%), followed by tube well irrigated (3–20%) (Figure 3). Healthy plants had higher antagonistic bacteria (12–30%), followed by that of diseased plants (1–20%) (Figure 4). The distribution of percent antagonistic bacteria in different plant parts was also variable. The highest number of antagonistic bacteria were observed in rhizosphere (23%), followed by that of root (14%) and leaf endospheres (9%), respectively.

### Identification and Phylogenetic Lineage of Antagonistic Bacteria

**FIGURE 5:**
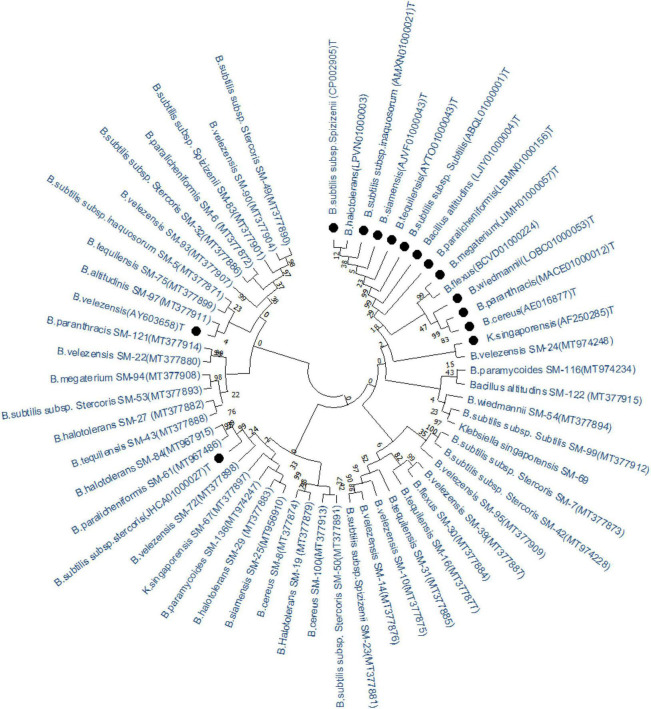
Phylogenetic lineages of antagonistic bacteria based on 16S rRNA gene. Tree leaves represent bacterial genus with accession numbers and likely top hit strain, isolated in this study. Type strains downloaded from NCBI are represented with black dots (•).

Based on 16S rRNA gene analysis, the antagonistic bacteria showing ≥48% antagonism were identified as *Bacillus* spp. except for the strain SM-67 and SM-69 which belonged to *klebsiella* spp., respectively. The homology with type strains, identity, and 16S rRNA accession number is given in Table 2. The phylogenetic lineage based on 16S rRNA gene is given in Figure 5. The optimal tree with the sum of branch length = 168.4 is shown in Figure 5. *Bacillus* spp. strain SM-7 showed the highest bootstrap value of 100. The percentage of replicated phylogenetic tree in which the associated taxa are grouped in the bootstrap test (500 replicates) is shown next to the branches of the tree (Figure 5). Type strains are represented with black dots in front of them, while identified strains are represented with their code names. Phylogenetic lineage was calculated based on the 16S rRNA nucleotide sequences of different strains.

**TABLE 2:** Identification of the antagonistic bacteria based on 16S rRNA gene analysis.

Identified strain	Inhibition^1^ (%)	Similarity^2^ (%)	Accession no.	Identified strain	Inhibition^1^ (%)	Similarity^2^ (%)	Accession no.
*B. subtilis* SM-5	54	99.7	MT377871	*B. subtilis* SM-50	53	99.2	MT377891
*B. paralicheniformis* SM-6	49	96.7	MT377872	*B. subtilis* SM-53	50	98.4	MT377893
*B. subtilis* SM-7	56	99.9	MT377873	*B. wiedmannii* SM-54	52	98.4	MT377894
*B. cereus* SM-8	48	99.9	MT377874	*B. paralicheniformis* SM-61	52	98.5	MT967486
*B. velezensis* SM-10	56	99.6	MT377875	*Klebsiella singaporensis* SM-67	50	96.1	MT377896
*B. velezensis* SM-14	52	94.5	MT377876	*K. singaporensis* SM-69	51	96.1	MT377897
*B. tequilensis* SM-16	51	91.6	MT377877	*B. velezensis* SM-72	58	99.8	MT377898
*B. halotolerans* SM-19	51	99.4	MT377879	*B. tequilensis* SM-75	50	99.7	MT377899
*B. velezensis* SM-22	49	96.7	MT377880	*B. subtilis* SM-83	53	93.5	MT377901
*B. subtilis* SM-23	53	96.5	MT377881	*B. halotolerans* SM-84	53	96.1	MT967915
*B. velezensis* SM-24	57	73.6	MT974248	*B. velezensis* SM-90	59	99.1	MT377904
*B. siamensis* SM-25	54	74.9	MT956910	*B. cabrialesii* SM-93	63	98.1	MT377907
*B. halotolerans* SM-27	49	96.3	MT377882	*B. megaterium* SM-94	54	99.4	MT377908
*B. halotolerans* SM-29	57	99.4	MT377883	*B. velezensis* SM-95	54	99.3	MT377909
*B. flexus* SM-30	49	93.8	MT377884	*B. altitudinis* SM-97	56	99	MT377911
*B. tequilensis* SM-31	51	91.8	MT377885	*B. subtilis* SM-99	54	99.6	MT377912
*B. subtilis* SM-32	54	99.2	MT377886	*B. cereus* SM-100	49	99	MT377913
*B. velezensis* SM-39	67	99.6	MT377887	*B. paramycoides* SM-116	57	87.6	MT974234
*B. subtilis* SM-42	49	99.9	MT974228	*B. paranthracis* SM-121	52	98.4	MT377914
*B. tequilensis* SM-43	53	99.8	MT377888	*B. altitudinis* SM-122	57	99.93	MT377915
*B. subtilis* SM-49	55	99.7	MT377890	*B. paramycoides* SM-136	48	99.6	MT974247

*B, Bacillus spp.*

*^1^Mean inhibition against four root rot pathogens, i.e., Fusarium moniliforme, Fusarium oxysporum, Macrophomina phaseolina, and Rhizoctonia solani.*

*^2^Similarity (%) of bacterial strains with closest EZ-taxon e-match.*

### Biocontrol Efficacy of Rhizobacteria Against Wheat Root Rot

**FIGURE 6:**
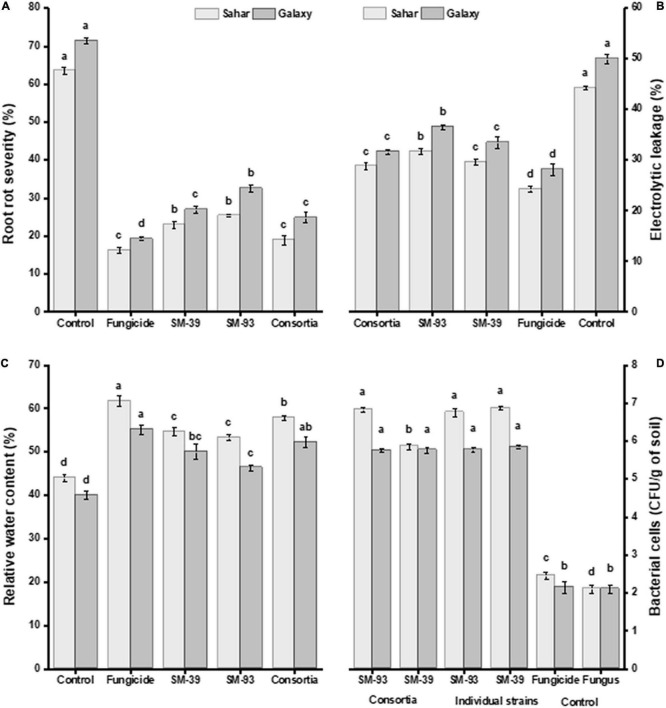
Effect of antagonistic bacteria on wheat challenged with *F. moniliforme.* **(A)** Root rot severity (%), **(B)** electrolytic leakage (%), **(C)** relative water content (%), and **(D)** colonization of bacteria to wheat rhizosphere. The values are the mean of three replications and having different letters within the same bar are significantly different from each other according to Fischer’s LSD test at *p* ≤ 0.05.

Inoculated *Bacillus* spp. significantly reduced *Fusarium* root rot severity in both wheat varieties, i.e., Sahar and Galaxy, as compared to uninoculated. A significant disease suppression over control was observed in the plants treated with consortium of *B. velezensis* SM-39 and *B. cabrialesii* SM-93 (52–62%) followed by that of *B. velezensis* SM-39 (49%) and *B. cabrialesii* SM-93 strain (42–49%) (Figure 6A). A similar trend in the performance of all treatments was observed in electrolytic leakage and relative water content (Figures 6B,C).

### Root Colonization

Antagonistic strains *B. velezensis* (SM-39) and *B. cabrialesii* (SM-93) considerably colonized the wheat roots either inoculated individually or in consortium (Figure 6D). Maximum root colonization was observed in *B. velezensis* treated plants (6.6–9.7 log CFU/g of rhizospheric soil), followed by that of *B. cabrialesii* (5.2–6.9 log CFU/g of rhizospheric soil) as shown in Table 3. A significant correlation was observed between the biochemical traits, root colonization, and disease severity (Table 4). Wheat variety had a significant effect on disease incidence and biochemical parameters (Supplementary Table 2).

**TABLE 3:** Habitat characteristics and root colonization of the *Bacillus* spp. exhibiting *in planta* biocontrol activity against wheat root rot.

Strain	*B. velezensis* SM-39	*B. cabrialesii* SM-93
Location	Gujranwala	Lahore
Irrigation source	Canal	Tube well
Plant health	Healthy	Healthy
Plant part	Rhizosphere	Root endosphere
Root colonization	6.6–9.7 log CFU^–1^	5.2–6.9 log CFU^–1^
% inhibition	49	42

*Plant health, plant health was determined based on phenotypic traits of wheat; CFU/g, colony forming unit/g of rhizospheric soil; % inhibition, % suppression of wheat root rot fungi.*

**TABLE 4:** Pearson correlations between disease severity, biochemical traits, and root colonization of antagonistic bacteria.

Treatment	EL (%)	RWC (%)	DS (%)
**Sahar**			
RWC	0.7418		
DS	0.8747*	0.3499	
RC	–0.3998	–0.2205	−0.5601
**Galaxy**			
RWC	0.7471		
DS	0.8989*	0.3968	
RC	–0.4016	–0.1466	−0.5661

EL, electrolytic leakage; RWC, relative water content; DS, disease severity; RC, root colonization; and * = p ≤ 0.05.

## Discussion

Antagonistic bacteria inhibit phytopathogens to protect the plants from various diseases (Schlemper et al., 2018). The diversity of antagonistic bacteria in the root rhizosphere and endosphere depends on various factors such as cropping system, cultivar, different agricultural practices, and climatic conditions (Na et al., 2019). In this study, antagonistic *Bacillus* spp. were found to be the most prevalent in wheat grown in the wheat-rice cropping system.

In wheat, rhizospheric isolates were found higher in number as compared to that of root and leaf endophytes. The higher rhizosphere population may be due to the rich nutrition of the rhizosphere where the plant secretes numerous secondary metabolites including the chemoattractants of the rhizobacteria (Truyens et al., 2015). These results are also in accordance with the earlier studies in which the root rhizosphere was considered as the hub of microbes. The less endophytic population of bacteria may be due to their inability to infiltrate the internal plant tissues. Endophytic bacteria infiltrate the plant’s internal tissues through active and passive mechanisms. The active mechanism involves chemotaxis while the passive involves the movement of bacteria through water flow from injured plant tissues (Cha et al., 2016).

A higher proportion of the bacteria obtained from wheat rhizosphere/endosphere was found to be antagonistic to either one or more pathogens causing wheat root rot. This distribution of antagonistic rhizobacteria in the wheat rhizosphere/endosphere could be due to the disease-suppressive soil of the wheat-rice cropping system (Hu et al., 2018). Another reason behind the abundance of the antagonists could be the specific bacterial community that had adapted this habitat through their antagonistic behavior, which was proved in our further findings, i.e., 16S rRNA identification. The bacterial antagonism was found to be highly variable and dependent on the type of pathogen. This change in antagonistic properties of different bacterial strains is due to their genetic makeup, production of secondary metabolites, plant health, and competition with other soil microbes and type of targeted pathogen (van der Voort et al., 2016). The distribution of antagonistic bacteria was also affected by different parameters such as crop locations, crop health, irrigation source, and plant parts, i.e., rhizosphere, root, and leaf endospheres. Antagonistic bacteria produce different secondary metabolites such as antibiotics and as a result, inhibit various phytopathogens. This antagonism could be due to competition of nutrients by activating the defense mechanism of the host plant and by the production of antifungal compounds, which play an important role in the biocontrol of phytopathogens. Antibiosis and competition are the main mechanisms through which *Bacillus* spp. control different diseases (Cawoy et al., 2015). *Bacillus* spp. produce different secondary metabolites such as surfactin, iturin, fengycin, and bacillomycins (Yoon et al., 2017).

The 16S rRNA gene analysis and homology with that of type strain available at EZ Bio cloud database revealed that bacteria obtained from wheat-rice cropping system and exhibiting significant antagonism were *Bacillus* spp. except for a single strain of *Pseudomonas* spp. and two strains of *Klebsiella* spp. The 16S rRNA gene sequence analysis is believed to be an excellent technique for bacterial identification and EzBioCloud is an updated 16S rRNA gene sequences database in line with the latest taxonomic changes (Zhou et al., 2016).

The phylogenetic tree was constructed for all the antagonistic strains based on the 16S rRNA gene sequence on MEGA X software (Afzal et al., 2019). It is an important tool to determine the evolutionary relationship within the strains for analyzing the variation at the genus level (Afzal et al., 2019). The antagonistic bacteria showed variable phylogenetic lineage based on 16S rRNA gene. Our findings of the prevalence of antagonistic *Bacillus* spp. are in accordance with the earlier studies (Earl et al., 2007). The ubiquitous distribution of *Bacillus* spp. in the rhizosphere may be due to their unique characteristics such as adaptability to the harsh environment through desiccation tolerance and ability to regenerate through sporulation (Joshi et al., 2013). The occurrence of wheat-associated antagonistic *Bacillus* spp. in the wheat-rice cropping system may also be due to the specific cropping pattern and its soil environment conditions (Kumar et al., 2012).

Wheat and rice belong to generate family and secrete common root exudates, namely, benzoxazinoids, phenolic acids, and scopoletin (Zahid, 2015). The abundance of *Bacillus* spp. in this cropping system may also be due to the root exudates mediated recruitment of specific microbes (Winter et al., 2019). The role of root exudates in shaping the specific rhizospheric community is well documented (Lv et al., 2016). *Bacillus* spp., being facultative anaerobe/aerobe, may also have higher survival in the rice grown in submerged conditions. The long-standing water creates anaerobic conditions which is an environment for the adaptation of specific microbial communities like *Bacillus* spp. The effect of waterlogging conditions on change in the microbial community is well documented (Mentzer et al., 2006). In waterlogging conditions, oxygen quantity decreases which leads to a reduction in respiration rate and activity of the microbial community. This in turn leads to variations in the distribution of the microbial community (Fuchslueger et al., 2014). Thus, the wheat-rice cropping system could develop disease suppressive soil and wheat grown in such a system could be a potential habitat of the antagonistic *Bacillus* spp.

*Bacillus* spp. with the best *in vitro* antagonistic abilities also controlled *Fusarium* root rot in net house conditions. These findings are in accordance with earlier reports, in which rhizospheric bacteria have successfully colonized the wheat rhizosphere (Ullah et al., 2020). However, in the current study, the strains also retained their population upon their inoculation as consortia. The biocontrol efficacy of antagonistic bacteria against *Fusarium* root rot is well understood (Winter et al., 2019). They adopt multiple mechanisms in plant defense including induction of defense enzymes against biotic and abiotic stress (Khanna et al., 2019c,d). In consortia, our bacterial strains showed the best activity against wheat root rot as compared to the individual ones. Their effect was at par with that of earlier reports in which efficacy of *Pseudomonas* spp. associated with monoculture wheat was assessed against root rot (Ullah et al., 2020). Our results are also advocated by Lounaci et al. (2016) findings, in which rhizospheric bacteria has efficiently controlled root rot severity. Fungicides also effectively control root rot of wheat and barley, but their irrational use poses a serious threat to the environment. These results indicate that antagonistic bacterial strains could be an ideal candidate for the biocontrol of *Fusarium* root rot disease (Ngumbi and Kloepper, 2016). Based on their disease suppression abilities, they could be an excellent substitute to the chemicals, i.e., fungicides, which have serious health implications on humans and eco-systems due to their excessive usage (Upadhyay et al., 2018).

The current study states that different rhizospheric and endospheric bacteria isolated from wheat-rice cropping system of Punjab, Pakistan and identified as *Bacillus* spp. displayed tremendous ability to inhibit different wheat pathogens such as *F. oxysporum*, *F. moniliforme*, *R. solani*, and *M. phaseolina*. So, it can be concluded that the continuous practice of wheat-rice cropping system could develop disease suppressive soil in certain fields and wheat grown in such fields could be a potential habitat of the antagonistic *Bacillus* spp.

## Data Availability Statement

The original contributions presented in the study are included in the article/Supplementary Material, further inquiries can be directed to the corresponding author/s.

## Author Contributions

ShM: methods, experiments, writing original draft, data analysis, and review and editing. AW: methodology, experiments, review and editing, and data curation. HY: data analysis, review and editing, methodology, and resources. MH: conceptualization, supervision, resources, funding, and review and editing. SaM: methodology, data analysis, and review and editing. HAE: funding, resourced, and review and editing. NK: analysis, software, and review and editing. All authors contributed to the article and approved the submitted version.

## Conflict of Interest

The authors declare that the research was conducted in the absence of any commercial or financial relationships that could be construed as a potential conflict of interest.

## Publisher’s Note

All claims expressed in this article are solely those of the authors and do not necessarily represent those of their affiliated organizations, or those of the publisher, the editors and the reviewers. Any product that may be evaluated in this article, or claim that may be made by its manufacturer, is not guaranteed or endorsed by the publisher.

## Footnotes

1
https://www.ezbiocloud.net/

2
https://submit.ncbi.nlm.nih.gov/

3
https://www.ezbiocloud.net/
