# Supplementary material for: Prevalence of Wheat Associated Bacillus spp. and Their Bio-Control Efficacy Against Fusarium Root Rot
Source: Front Microbiol. 2022 Mar 3;12:798619. doi: 10.3389/fmicb.2021.798619 (PMC8927631; doi:10.3389/fmicb.2021.798619)
Supplement: Supplementary file 1 [file Table_1.docx]

| **Strains** | ***F. moniliforme*** | ***F. oxysporum*** | ***R.solani*** | ***M.phaseolina*** | **Mean** | **Strain** | ***F. moniliforme*** | ***F. oxysporum*** | ***R.solani*** | ***M.phaseolina*** | **Mean**  **Inhibition** |
| --- | --- | --- | --- | --- | --- | --- | --- | --- | --- | --- | --- |
| SM-1 | 33.3±0.3 s | - | - | 45.2±0.1o | 19.6 | SM-22 | 38.0±0.3q | 47.6±0.2l | 61.9±0.1h | 50±0kl | 49.4 |
| SM-2 | - | - | 61.9±0.3h | - | 15.4 | SM-23 | 42.8±0.1n | 45.2±0.1nop | 66.6±0.2f | 57.1±0.5g | 52.9 |
| SM-3 | - | - | 45.2±0.1n | - | 11.3 | SM-24 | 57.1±0.1d | 47.6±0.5l | 66.6±0.3f | 57.1±0.1g | 57.1 |
| SM-5 | 40.4±0.1 op | 59.5±0.2e | 64.2±0.2g | 52.3±0.3j | 54.1 | SM-25 | 47.6±0.2j | 47.6±0.3l | 66.6±0.2f | 54.7±0.1hi | 54.1 |
| SM-6 | - | 73.8±0.4a | 71.4±0.1b | 52.3±0.4j | 49.4 | SM-27 | 35.7±0.3r | 45.2±0.1nop | 66.6±0.4f | 47.6±0.2n | 48.8 |
| SM-7 | 42.8±0.1n | 54.7±0.2fg | 69.0±0.1cd | 57.1±0.1g | 55.9 | SM-28 | - | 52.3±0.3l | 69.0±0.1cd | 50±0kl | 42.8 |
| SM-8 | 47.6±0.2i | 38.0±0.1v | 64.2±0.3g | 40.4±0.3q | 47.6 | SM-29 | 52.3±0.1f | 52.3±0.2i | 64.2±0.4g | 59.5±0.2f | 57.1 |
| SM-10 | 50±0.3g | 52.3±0.2i | 66.6±0.2f | 54.7±0.1hi | 55.9 | SM-30 | 47.6±0.5i | 40.4±0.8r | 57.1±0.1j | 50±0.3k | 48.8 |
| SM-11 | - | - | 59.5±0.3i | - | 14.8 | SM-31 | 50±0.1g | 40.4±1.0rs | 66.6±0.4f | 47.6±0.2n | 51.1 |
| SM-12 | - | - | 47.6±0.2m | - | 11.9 | SM-32 | 50±0.3g | 45.2±0.2no | 66.6±0.2f | 54.7±0.3i | 54.1 |
| SM-13 | 30.9±0.1t | 42.8±0.2q | 57.1±0.2j | 50±0kl | 45.2 | SM-33 | - | 38.0±0.7u | 57.1±0.1j | 42.8±0.1p | 34.5 |
| SM-14 | 42.8±0.3n | 47.6±0.1l | 66.6±0.4f | 52.3±0.1j | 52.3 | SM-34 | - | 50±ok | 45.2±0.1n | - | 23.8 |
| SM-15 | - | 40.4±0.1t | 71.4±0.1b | 52.3±0.2j | 41.0 | SM-36 | - | - | 64.2±0.2g | 47.6±0.2n | 27.9 |
| SM-16 | 42.8±0.1n | 42.8±0.2q | 64.2±0.2g | 54.7±0.1hi | 51.1 | SM-37 | 38.0±0.1q | 42.8±0.16q | 59.5±0.2i | 42.8±0.7p | 45.8 |
| SM-17 | 40.4±0.3op | - | 50±0.1l | - | 22.6 | SM-38 | 50±0.2g | - | 42.8±0.1o | - | 23.2 |
| SM-18 | 38.0±0.2q | 38.0±0.4uv | 57.1±0.1j | 40.4±0.4q | 43.4 | SM-39 | 61.9±0.1b | 64.2±0.2c | 73.8±0.3a | 66.6±0.2b | 66.6 |
| SM-19 | 45.2±0.1lm | 42.8±0.1q | 66.6±0.4f | 50±0kl | 51.1 | SM-40 | 40.4±0.2o | 47.6±0.2l | 52.3±0.1k | 50±0.3kl | 47.6 |
| SM-20 | 35.7±0.4r | - | - | - | 8.9 | SM-42 | 45.2±0.1lm | 45.2±0.1nop | 61.9±0.1h | 45.2±0.6o | 49.3 |
| SM-21 | - | 45.2±0.2p | 50±0.1l | 50±0.3kl | 36.3 | SM-43 | 42.8±0.1n | 50±0.3jk | 66.6±0.1f | 52.3±0.6j | 52.9 |

**Table: S1** Percent inhibition of different root rot pathogens by wheat associated bacteria

The values are mean of three replications and having different letters within same bar are significantly different from each other according to Fischer’s LSD test at p≤ 0.05., F=*Fusarium*, R= *Rhizoctonia M* =*Macrophomina,* - = non antagonistic

| **Strains** | ***F. moniliforme*** | ***F. oxysporum*** | ***R.solani*** | ***M.phaseolina*** | **Mean** | **Strain** | ***F. moniliforme*** | ***F. oxysporum*** | ***R.solani*** | ***M.phaseolina*** | **Mean**  **Inhibition** |
| --- | --- | --- | --- | --- | --- | --- | --- | --- | --- | --- | --- |
| SM-44 | - | - | 35.7±0.4q | - | 8.9 | SM-64 | - | 38.0±0.4u | 61.9±0.1h | 40.4±0.5q | 35.1 |
| SM-45 | - | 50±0k | 57.1±0.3j | 42.8±0.1p | 37.4 | SM-65 | - | 40.4±0.3t | 61.9±0.1h | 35.7±0.1r | 34.5 |
| SM-46 | - | 47.6±0.2l | 61.9±0.2h | 50±0kl | 39.8 | SM-66 | 40.4±0.2p | 40.4±0.4t | 66.6±0.2f | 40.4±0.3q | 47.0 |
| SM-47 | - | 52.3±0.1i | 69.0±0.3cd | 57.1±0.1g | 44.6 | SM-67 | 42.8±0.1n | 50±0.2jk | 66.6±0.2f | 45.2±0.1o | 49.9 |
| SM-48 | - | 33.3±0.1x | - | - | 8.3 | SM-68 | - | 42.8±0.3q | 59.5±0.3i | 52.3±0.3j | 38.6 |
| SM-49 | 47.6±0.1i | 50±0.6j | 66.6±0.3f | 57.1±0.1g | 55.3 | SM-69 | 45.2±0.1m | 42.8±0.1q | 61.9±0.1h | 52.3±0.2j | 50.5 |
| SM-50 | 47.6±0.2i | 54.7±0.3f | 59.5±0.2i | 50±0.3k | 52.9 | SM-70 | 42.8±0.1n | 33.3±0.2x | 42.8±0.1o | 50±0.6k | 42.2 |
| SM-51 | - | 38.0±0.4uv | 52.3±0.1k | - | 22.6 | SM-72 | 57.1±0.4d | 50±0k | 73.8±0.2a | 52.3±0.4j | 58.3 |
| SM-52 | 40.4±0.2op | 40.4±0.2t | 64.2±0.2g | 45.2±0.1o | 47.6 | SM-73 | - | 50±0.4j | 71.4±0.1b | 52.3±0.1j | 43.4 |
| SM-53 | 42.8±0.2n | 47.6±0.4l | 57.1±0.3j | 52.3±0.1j | 49.9 | SM-75 | 42.8±0.1n | 45.2±0.1nop | 61.9±0.2h | 50±0kl | 49.9 |
| SM-54 | 47.6±0.2i | 45.2±0.5no | 66.6±0.2f | 50±0kl | 52.3 | SM-77 | - | - | 30.9±0.3r | - | 7.7 |
| SM-55 | 45.2±0.1kl | - | 28.5±3.0r | - | 18.4 | SM-82 | - | - | 45.2±0.1n | - | 11.3 |
| SM-57 | - | 40.4±0.9t | 57.1±0.5j | 46.6±0.4n | 36.0 | SM-83 | 45.2±0.3kl | 45.2±0.3nop | 66.6±0.4f | 54.7±0.2i | 52.9 |
| SM-58 | - | - | - | 66.6±0.7b | 16.6 | SM-84 | 45.2±0.1lm | 50±0k | 59.5±0.2i | 57.1±0.1g | 52.9 |
| SM-59 | - | 47.6±0.3l | 61.9±0.3h | 55.5±0.2h | 41.2 | SM-85 | - | 40.4±0.1st | 52.3±0.1k | 35.7±0.5r | 32.1 |
| SM-60 | - | 42.8±0.1q | 59.5±0.3i | 48.8±0.1lm | 37.8 | SM-86 | - | 59.5±0.2e | 69.0±0.4c | 47.6±0.2mn | 44.0 |
| SM-61 | 42.8±0.1n | 52.3±0.1i | 64.2±0.3g | 50±0.3kl | 52.3 | SM-87 | 59.5±0.2c | - | - | - | 14.8 |
| SM-62 | 35.7±0.2r | 35.7±0.2v | 57.1±0.1j | 40.4±0.6q | 42.2 | SM-88 | 38.0±0.3q | 30.9±0.7y | 52.3±0.2k | 45.2±0.5o | 41.6 |
| SM-63 | 42.8±0.4n | 40.4±0.3st | 69.0±0.1d | 47.6±0.2n | 49.9 | SM-89 | - | 44.4±0.3p | 60±0i | - | 26.1 |

**Table: S1** (Continued)Percent inhibition of different root rot pathogens by wheat associated bacteria

**Table: S1**(Continued)Percent inhibition of different root rot pathogens by wheat associated bacteria

| **Strains** | ***F. moniliforme*** | ***F. oxysporum*** | ***R. solani*** | ***M. phaseolina*** | **Mean** | **Strain** | ***F. moniliforme*** | ***F. oxysporum*** | ***R. solani*** | ***M. phaseolina*** | **Mean Inhibition** |
| --- | --- | --- | --- | --- | --- | --- | --- | --- | --- | --- | --- |
| SM-90 | 44.4±0.3m | 62.2±0.4d | 68.8±0.2cd | 61.9±0.3e | 59.1 | SM-111 | - | 54.7±0.1g | 66.6±0.2f | 57.1±0.1g | 44.6 |
| SM-91 | - | 66.6±0.2b | - | - | 16.6 | SM-112 | 54.7±0.2e | 50±0.4jk | - | 59.5±0.2f | 41.0 |
| SM-92 | - | 33.3±0.3x | - | - | 8.3 | SM-114 | - | - | 45.2±0.1n | - | 11.3 |
| SM-93 | 64.4±0.2a | 55.5±0.3f | 68.8±1.3de | 64.2±0.1d | 63.2 | SM-116 | 59.5±0.3c | 52.3±0.3i | 64.2±0.2g | 52.3±0.1j | 57.1 |
| SM-94 | 44.4±0.3lm | 53.3±0.4h |  | 69.0±3.3c | 54.2 | SM-117 | 50±0.1g | - | - | - | 12.5 |
| SM-95 | 44.4±0.3m | 53.3±0.3h | 64.4±0.4g | 54.7±0.2hi | 54.2 | SM-120 | 33.3±0.2s | - | 40.4±0.4p | - | 18.4 |
| SM-96 | - | 46.6±0.2m | - | - | 11.6 | SM-121 | 48.8±0.6h | 47.6±0.3l | 61.9±0.2h | 50±0.3kl | 52.0 |
| SM-97 | 42.8±0.1n | 52.3±0.1i | 66.6±0.3f | 61.9±0.3e | 55.9 | SM-122 | 62.2±0.4b | 50±0k | 64.2±0.1g | 52.3±0.1j | 57.2 |
| SM-98 | - | 50±0k | 57.1±0.6j | 59.5±0.2f | 41.6 | SM-124 | 30.9±0.2t | - | 30.9±0.1r | - | 15.4 |
| SM-99 | 54.7±0.2e | 35.7±0.2w | 64.2±0.3g | 61.9±0.1e | 54.1 | SM-125 | 45.2±0.1n | - | 66.6±0.3f | 59.5±0.2f | 42.8 |
| SM-100 | - | 61.9±0.3d | 64.2±0.4g | 71.4±0.1a | 49.4 | SM-127 | - | 45.2±0.5n | 66.6±0.4ef | 52.3±0.1j | 41.0 |
| SM-101 | - | 42.8±0.1q | 50±0l | - | 23.2 | SM-128 | - | 45.2±0.3n | 52.3±0.5k | - | 24.4 |
| SM-102 | - | 47.6±0.2l | 42.8±0.3o | - | 22.6 | SM-129 | - | - | - | 42.8±0.1p | 10.7 |
| SM-103 | - | 28.5±0.5z | 42.8±0.1o | - | 22.6 | SM-130 | - | 38.0±0.3u | 64.2±0.2g | 42.8±0.1p | 36.3 |
| SM-110 | 42.8±0.1n | 54.7±0.2fg | - | 54.7±0.1hi | 38.6 | SM-136 | 64.2±0.8a | 59.5±0.3e | 69.0±0.1cd | - | 48.2 |

The values are mean of three replications and having different letters within same bar are significantly different from each other according to Fischer’s LSD test at p≤ 0.05., F=*Fusarium*, R= *Rhizoctonia M* =*Macrophomina,* - = non antagonistic
