# Supplementary material for: Prevalence of Wheat Associated Bacillus spp. and Their Bio-Control Efficacy Against Fusarium Root Rot
Source: Front Microbiol. 2022 Mar 3;12:798619. doi: 10.3389/fmicb.2021.798619 (PMC8927631; doi:10.3389/fmicb.2021.798619)
Supplement: Supplementary file 2 [file Table_2.docx]

**Table: S2 Analysis of variance (ANOVA) for *Fusarium* root rot disease, electrolytic leakage, relevant water content and bacterial colonization of wheat.**

| **Variables** | **Source** | **DF** | **SS** | **MS** | **F** | **P value** |
| --- | --- | --- | --- | --- | --- | --- |
|  |  |  |  |  |  |  |
| DS | Treatment | 4 | 9739.6 | 2434.91 | 1096.30 | <0.001 |
|  | Variety | 1 | 235.2 | 235.20 | 105.90 | <0.001 |
|  | Treatment x Variety | 4 | 27.1 | 6.77 | 3.05 | 0.0440 |
|  | Error | 18 | 40.0 | 2.22 |  |  |
|  | Total | 27 | 10051.4 |  |  |  |
| EL | Treatment | 4 | 1515.38 | 378.845 | 217.49 | <0.001 |
|  | Variety | 1 | 134.75 | 134.747 | 77.36 | <0.001 |
|  | Treatment x Variety | 4 | 7.67 | 1.916 | 1.10 | 0.3867 |
|  | Error | 18 | 31.35 | 1.742 |  |  |
|  | Total | 27 | 1691.14 |  |  |  |
| RWC | Treatment | 4 | 927.36 | 231.840 | 79.01 | <0.001 |
|  | Variety | 1 | 232.41 | 232.408 | 79.20 | <0.001 |
|  | Treatment x Variety | 4 | 9.21 | 2.303 | 0.78 | 0.5497 |
|  | Error | 18 | 52.82 | 2.934 |  |  |
|  | Total | 27 | 1224.76 |  |  |  |
| RC | Treatment | 5 | 0.493 | 0.0986 | 0.91 | 0.4909 |
|  | Variety | 1 | 2.986 | 2.9858 | 27.65 | <0.001 |
|  | Treatment x Variety | 5 | 0.419 | 0.0839 | 0.78 | 0.5769 |
|  | Error | 22 | 2.376 | 0.1080 |  |  |
|  | Total | 33 | 133.468 |  |  |  |
| DF = degree of freedom, SS= sum of squares MS = mean of squares, DS= disease severity, EL= electrolytic leakage, RWC= relative water content, RC= root colonization. | | | | | | |
